# An *in vitro* comparison of three nucleus pulposus removal techniques for partial intervertebral disc replacement: An ultra‐high resolution MRI study

**DOI:** 10.1002/jsp2.1232

**Published:** 2022-12-12

**Authors:** Tamanna Rahman, Nicoleta Baxan, Robert T. Murray, Saman Tavana, Thomas P. Schaer, Nigel Smith, Jonathan Bull, Nicolas Newell

**Affiliations:** ^1^ Biomechanics Group, Department of Mechanical Engineering Imperial College London London UK; ^2^ Department of Bioengineering Imperial College London London UK; ^3^ Biological Imaging Centre, Central Biomedical Services Imperial College London, Hammersmith Hospital Campus London UK; ^4^ Femtosecond Optics Group, Blackett Laboratory, Department of Physics Imperial College London London UK; ^5^ Department of Clinical Studies, School of Veterinary Medicine, New Bolton Center University of Pennsylvania Philadelphia Pennsylvania USA; ^6^ Division of Surgery and Interventional Science University College London Stanmore UK; ^7^ Department of Neurosurgery BARTS Health NHS Trust London UK

**Keywords:** intervertebral disc biomechanics, nuclectomy, nucleus replacement surgery

## Abstract

**Background:**

Nuclectomy, also known as nucleotomy, is a percutaneous surgical procedure performed to remove nucleus material from the center of the disc. Multiple techniques have been considered to perform a nuclectomy, however, the advantages and disadvantages of each are not well understood.

**Aims:**

This *in vitro* biomechanical investigation on human cadaveric specimens aimed to quantitatively compare three nuclectomy techniques performed using an automated shaver, rongeurs, and laser.

**Material & Methods:**

Comparisons were made in terms of mass, volume and location of material removal, changes in disc height, and stiffness. Fifteen vertebra–disc–vertebra lumbar specimens were acquired from six donors (40 ± 13 years) and split into three groups. Before and after nucleotomy axial mechanical tests were performed and T2‐weighted 9.4T MRIs were acquired for each specimen.

**Results:**

When using the automated shaver and rongeurs similar volumes of disc material were removed (2.51 ± 1.10% and 2.76 ± 1.39% of the total disc volume, respectively), while considerably less material was removed using the laser (0.12 ± 0.07%). Nuclectomy using the automated shaver and rongeurs significantly reduced the toe‐region stiffness (p = 0.036), while the reduction in the linear region stiffness was significant only for the rongeurs group (p = 0.011). Post‐nuclectomy, 60% of the rongeurs group specimens showed changes in the endplate profile while 40% from the laser group showed subchondral marrow changes.

**Discussion:**

From the MRIs, homogeneous cavities were seen in the center of the disc when using the automated shaver. When using rongeurs, material was removed non‐homogeneously both from the nucleus and annulus regions. Laser ablation formed small and localized cavities suggesting that the technique is not suitable to remove large volumes of material unless it is developed and optimized for this application.

**Conclusion:**

The results demonstrate that both rongeurs and automated shavers can be used to remove large volumes of NP material but the reduced risk of collateral damage to surrounding tissues suggests that the automated shaver may be more suitable.

## Graphical abstract

Multiple techniques have been considered to perform a nuclectomy, however, the advantages and disadvantages of each are not well understood. This *in vitro* biomechanical investigation on human cadaveric specimens aimed to quantitatively compare three nuclectomy techniques performed using an automated shaver, rongeurs, and laser.

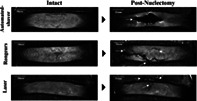

## 1 INTRODUCTION

Lower back pain (LBP) has been the most common cause of global disability since 1990^1^ affecting 70%–85% of people at some point in their lifetime.^2^ It is a multifactorial problem associated with multiple etiologies such as physical factors, social demographic characteristics, and psychosocial factors. Disc degeneration is considered to be one of the underlying factors that contribute to a patient developing LBP,^3^ and has therefore been the focus of numerous biomechanical studies.^4^ Spinal fusion is the current gold standard surgical treatment for severely degenerated discs, where pain is debilitating.^5^ Despite providing good short‐term effects, rigid spinal fixation can subject adjacent spinal segments to higher strains and stresses during daily activities,^6^, ^7^ and more than 30% of cases develop adjacent disc disease.^8^

As opposed to fusion, which is more appropriate for patients with severely degenerated discs,^9^ nucleus replacement or augmentation has been proposed as an early surgical treatment for younger patients with chronic LBP and moderate disc degeneration (disc height ≥5 mm).^9^, ^10^, ^11^ Surgically, the procedure is a relatively simple percutaneous technique that offers multiple revision options including total disc replacement and fusion in instances of unsuccessful outcomes.^9^

The main objective of a nucleus replacement surgery is to treat pain by substituting either partially or completely the degenerated nucleus pulposus (NP) tissue with a device that restores physiological disc height and biomechanics, while also slowing the degenerative cascade^12^, ^13^ and preserving the anatomical structure of the annulus fibrosus (AF). Prior to a NP replacement, disc material is removed in a process called nuclectomy or nucleotomy. Nuclectomy for a nucleus replacement surgery has similarities to the percutaneous nucleotomies or discectomies performed to treat disc herniation. Discectomies have been studied more widely due to their common clinical use.^14^, ^15^, ^16^, ^17^, ^18^, ^19^ Clinical studies have shown that discectomy with limited nucleus removal is associated with higher risk of reherniation, while more aggressive NP removal is associated with accelerated disc degeneration and increased risk of LBP.^17^, ^20^, ^21^, ^22^, ^23^ Furthermore, *in vitro* tests have demonstrated that nuclectomy alone, without NP replacement, alters disc biomechanics, including reducing internal pressures, increasing the range of the neutral zone, and changing internal strain distributions.^24^, ^25^, ^26^, ^27^, ^28^, ^29^, ^30^

Studies have also shown that nucleus replacement following nuclectomy can re‐establish compressive stabilization of the disc, restore the intact range of motion, and intradiscal pressure,^11^, ^31^, ^32^ therefore, showing the potential of this treatment. However, a current challenge with nucleus replacement surgery is preventing device expulsion or migration following implantation,^9^, ^33^ which may be due to insufficient NP removal, removal of material from regions other than the center of the disc before the replacement device is implanted, or a mechanically incompetent AF.

Various techniques can be implemented to remove NP material. These include simple methods such as using rongeurs,^34^, ^35^ or more advanced methods such as automated shavers or thermal techniques.^35^, ^36^ Thermal techniques such as radiofrequency or laser systems have been used to carry out lumbar disc decompression to treat patients with discogenic lumbar pain and sciatica.^37^, ^38^, ^39^, ^40^, ^41^, ^42^ Enzymatic approaches, such as using Chymopapain or Chondroitinase ABC (also called Condoliase), have been found to be effective in digesting NP material,^43^ however safety concerns have been raised regarding the use of Chymopapain.^37^, ^44^, ^45^ Condoliase has been predominantly used in animals^46^ although clinical trials are ongoing.^47^

Nucleus replacement devices have been designed to fill a homogenous void created at the center of an intervertebral disc (IVD).^48^, ^49^, ^50^, ^51^ Therefore, a successful nuclectomy should result in a uniform cavity formed at the center of the disc, with minimal collateral damage around the entry point through the AF such that its load‐bearing capacity is unaffected.

Previous studies have compared nuclectomy techniques using clinical MR images or transversely cutting the specimens to allow manual measurements of damage.^10^, ^37^, ^42^, ^52^ Since the volumes of material removal are relatively small, and intervertebral disc material is soft these techniques have limited ability to compare methods due to the challenges of segmenting voids on low‐resolution MRIs and the invasive nature of sectioning discs. The use of a 9.4T MRI scanner that captures high‐resolution images may allow these IVD changes to be quantified non‐invasively.

The aim of this study is to compare the efficacy of three different NP removal methods (an automated shaver, rongeurs, and laser) in terms of location and mass of material removed, extent of internal damage, and biomechanical changes using human cadaveric specimens. The results will improve our understanding of how different nuclectomy techniques affect both the internal structure and biomechanics of a disc. This information will be useful to implant designers and potentially allow surgeons to make more informed choices as to the most appropriate method to achieve nucleus removal while minimizing damage to surrounding tissues.

## 2 METHODS

### 2.1 Specimen preparation

**FIGURE 1:**
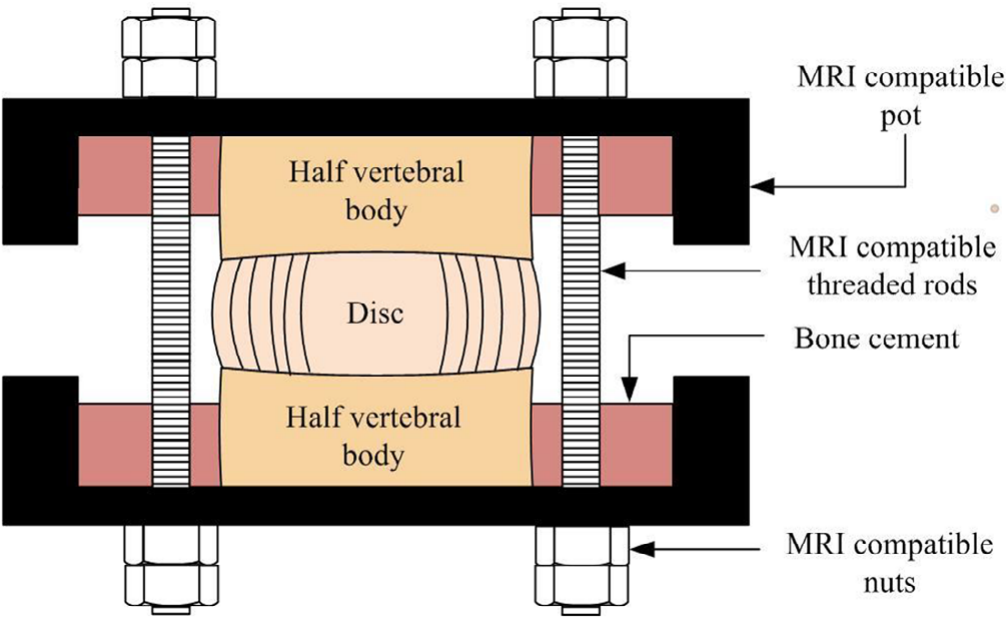
Sagittal view of vertebrae–disc–vertebra specimen potted within the MRI‐compatible pot with three nylon rods passing through the top and bottom pots. Note that the third nylon rod is not shown in the diagram. The position of the sample was fixed by tightening four nuts to each rod (two at the top pot and two at the bottom pot), to maintain a constant disc displacement while the MR images were captured.

Fifteen lumbar functional spinal units were harvested from six human cadaveric spines (5 male, 1 female), average age of 40 ± 13 years. Ethical approval was obtained from the Tissue Management Committee of the Imperial College Tissue Bank (ethical approval number: 17/WA/0161). Each vertebral body was transversely dissected into two halves. Facet joints and soft tissues were removed with care taken to not damage the IVD. Transverse processes were cut at a distance of approximately 2 mm from the edge of vertebral body to ensure that the specimen could fit within the 84.6 mm diameter bore of the MRI coil. The cranial and caudal vertebrae were fixed into position within MRI‐compatible pots using polymethyl‐methacrylate, while ensuring the mid‐transverse plane of the disc was parallel to the base of the pot. A bespoke MRI‐compatible rig, described in more detail by Tavana et al.,^53^ was used to maintain disc height during the scan time (Figure 1) and to ensure each specimen could be accurately and repeatedly placed within the bore of the scanner.

Specimens were stored at −20°C and before testing they were thawed overnight while being wrapped with phosphate‐buffered saline (PBS, 0.15 M) soaked tissue and double bagged to allow the discs to reach a steady state of hydration, and to equilibrate at room temperature. Throughout the preparation process, the specimens were regularly sprayed with PBS to maintain hydration. To compare the stiffnesses between intact and denucleated IVDs, specimens were axially compressed between 50 N and 1 kN at 1 Hz using a universal material testing machine (Instron, Model 5866, High Wycombe, UK). 50 N was chosen as it ensured contact between the pot and MRI‐compatible rig during the MRI scans. Additionally, 50 N has been suggested to represent the load on a lumbar IVD when lying prone.^54^ A peak load of 1kN was selected as it provides the average intradiscal pressure experienced when standing relaxed. The average of the *in vivo* intradiscal pressures reported by Wilke et al.,^54^ Sato et al.,^55^ and Takahashi et al.^56^ when standing relaxed is 586 ± 180 kPa. By converting this pressure to a load using the average cross‐sectional area of the specimens used in this study (1703 ± 128.4 mm^2^) a value of 995 N (~1 kN) was obtained. Loading at 1 Hz was used to approximately represent a step frequency when walking slowly.^57^

To ensure that stiffness data were reproducible, four cycles of testing were completed to precondition the specimen. Preliminary work showed that five cycles were sufficient to obtain a repeatable force‐displacement response (changes in stiffnesses between cycles within 1%, this is also in agreement with previous studies^58^, ^59^). Thus, data from the fifth cycle was used to calculate stiffnesses from the toe and linear regions of the loading portion of the compressive cycle. The toe‐region stiffness was calculated as the slope of the curve between 50 and 200 N, the higher bound was chosen to ensure that it captured most of the toe‐region before the loading entered a linear region. The linear region was calculated as the slope of the curve between 500 and 900 N of the loading portion of the compressive cycle. Following the fifth compressive cycle, the specimen was allowed to recover for 5 min before its displacement was fixed after a nominal load of 50 N had been applied (Figure 1). Preliminary work showed that following this loading, 5 min of rest was sufficient to recover more than 99% of the initial disc height. Longer rest periods would result in disc height recovery less than the resolution of the MR images that were subsequently captured.

### 2.2 Pre‐nuclectomy ultra‐high field MRI scans

Following mechanical testing, each specimen was MR imaged before nuclectomy using an ultra‐high field (9.4T) MRI system (Bruker BioSpec, Ettlingen, Germany). The Pfirrmann's grade^60^ of each specimen was assigned by three observers based on T2 MRIs (RARE sequence, coronal plane, spatial resolution = 90 × 90 μm^2^, slice thickness = 800 μm, 20 min scan time) and the average was taken. Using the 9.4T MRI to determine Pfirrmann's grade has been shown to be more reliable.^61^ Specimens were deemed suitable for this study if the MRIs showed no signs of endplate damage, AF tears, and degeneration grade was 4 or less and central disc height greater than 5 mm. When assigning specimens to the various treatment groups, it was ensured that all lumbar levels and degeneration grades were included in each group (Table 1). The intact specimens’ MRI provided a reference to which the post‐nuclectomy MRI scans were compared. Care was taken to ensure that specimen orientation remained consistent between pre‐ and post‐nuclectomy scans by using an alignment jig fitted to the bottom pot.

**TABLE 1:** Summary of the specimens used in this study

Donor No	Age (year)	Sex	Specimen No	Level	Pfirrmann grade	Nuclectomy technique
1	22	F	1	L2–L3	2	Rongeurs
			2	L4–L5	3	Auto‐shaver
2	38	M	3	L3–L4	3	Laser
			4	L4–L5	3	Rongeurs
3	42	M	5	L1–L2	2	Laser
			6	L2–L3	2	Auto‐shaver
4	40	M	7	L1–L2	2	Laser
5	36	M	8	L1–L2	2	Rongeurs
			9	L2–L3	3	Laser
			10	L3–L4	3	Auto‐shaver
			11	L4–L5	3	Laser
6	65	M	12	L1–L2	4	Auto‐shaver
			13	L2–L3	3	Rongeurs
			14	L3–L4	3	Rongeurs
			15	L4–L5	3	Auto‐shaver

Mimics (Materialise HQ, v.19.0, Leuven, 97 Belgium) was used to measure the central disc height from the coronal MRI slice with the largest lateral disc width on the corresponding axial slice. A horizontal line was drawn between the outermost aspects on both the left and right sides of the disc on the coronal view to determine the horizontal midpoint. Disc height was measured at the horizontal midpoint by selecting both the caudal and cranial boundaries between the disc and the endplates. Two further height measurements were made on the neighboring slices, with the average of the three measurements taken as the disc height.

### 2.3 Nuclectomy using the automated‐shaver

**FIGURE 2:**
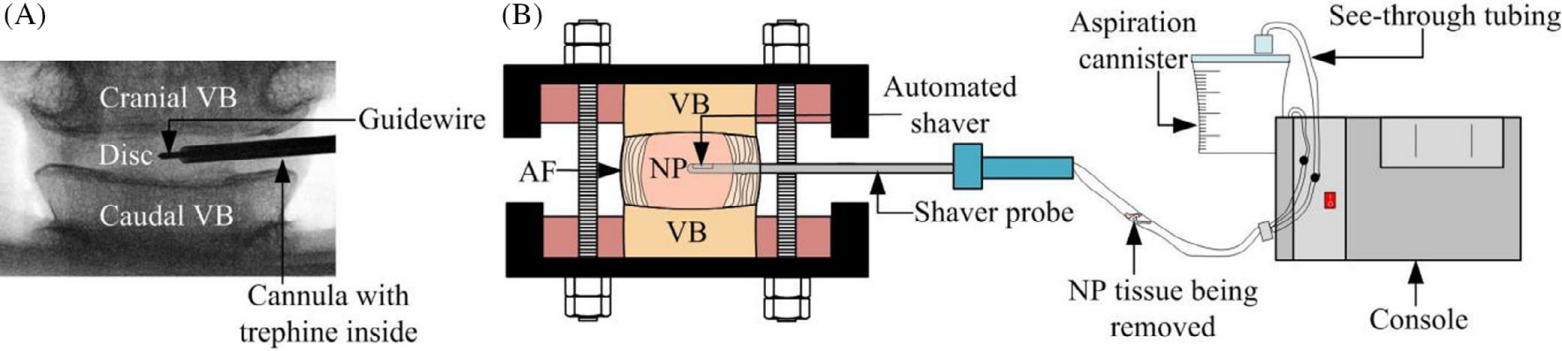
(A) Fluoroscopic coronal view image of the disc specimen following insertion of guidewire and cannula with trephine to confirm positioning. (B) The automated shaver is inserted into the disc space. The excised nucleus material can be observed as it travels through the transparent tubing to the aspiration canister. The removed material is collected in the aspiration canister along with the irrigation water

The automated shaver (Nucleotome®, Clarus Medical, Minneapolis, MN) consists of a 3 mm round‐tipped probe with an oscillating cutting mechanism augmented by a continuous lavage‐aspiration cycle via a side port at the tip. The probe is connected to a console with a fluid source and an aspiration system (Figure 2). A scalpel was used to make a 2 mm posterolateral annulotomy, a guidewire was then inserted through this incision. The guidewire's position was confirmed via C‐arm fluoroscopy (InsightFD Mini‐C‐Arm, Fluoroscan, MA). A trephine and cannula were inserted over the guidewire to incise the AF (Figure 2), the trephine was then replaced by the probe. As the probe was activated, the user was able to observe NP material being aspirated and then collected in the aspiration canister. The probe was moved within the disc space in a fan‐shaped pattern while being systematically rotated on its own axis to maximize the amount of NP material that could be removed. Particular attention was paid to the “shoulder” region to prevent a conical fan‐shaped nuclectomy. The “shoulder” region is the area adjacent to the automated‐shaver probe's entry site.

Previous clinical trials and studies have recommended using the probe within the disc space for 20 min.^11^, ^36^ However, preliminary investigation has shown that small amounts of disc material were still aspirated after 20 min. Therefore, in this study, the procedure was terminated when no visible amount of disc material was passing through the transparent tubing for a period of 2 min after the probe was in the disc space for 20 min (Figure 2). The NP material collected in the aspiration canister was filtered and weighed three times, and the dimensions of the AF insult post‐surgery were also measured to calculate the average ± SD.

### 2.4 Nuclectomy using rongeurs

A posterolateral triangular incision was made through the AF with a scalpel to enable the 3 mm diameter rongeurs to be inserted into the disc. NP material was torn away with the rongeurs, as they were withdrawn from the disc. The removed NP material was placed on preweighed filter paper. The procedure was terminated at the surgeon's discretion when no visible amount of material was being collected following three consecutive attempts at extracting NP material. Immediately following the surgery, the hydrated mass of removed NP material was weighed, and the height and width of the AF insult made by the rongeurs was measured three times using digital calipers.

### 2.5 Nuclectomy using laser

An 18‐gauge needle was inserted into the AF posterolaterally at the mid‐height of the disc. To ensure correct positioning, measurements were taken from axial slices of the intact MRIs to determine the target depth of needle insertion. A 400‐μm core optical fiber (Thorlabs FT400EMT) was mechanically stripped of the polymer coating at its tip and cleaned using isopropyl alcohol. The fiber tip was cleaved and was then dipped in iodine solution and carbonized before being inserted into the needle.^62^ Two position markers were drawn on the fiber to ensure that the fiber was positioned 5 mm beyond the needle‐tip to prevent localized needle heating and to ensure that the fiber was not protruding more than 15 mm beyond the needle‐tip and thus into AF tissue. Light from a continuous‐wave (CW) ytterbium fiber laser (IPG Photonics, 1070 nm) was coupled into the free end of the optical fiber. The 1070 nm wavelength of the laser was chosen to match previous studies that typically employed a 1064 nm CW Nd:YAG system. In line with the studies that have used laser ablation techniques to remove NP tissue, the laser system was calibrated to deliver 1 s duration pulses at 10 W followed by 4 s rest between pulses until a total of approximately 1200 J of energy had been delivered.^37^, ^39^, ^62^, ^63^, ^64^ The rest period allowed for heat dissipation. Every 400 J, the fiber was removed from the disc, re‐cleaved, carbonized, and then reinserted further into the disc to ensure maximum tissue removal.

Preliminary work showed that to achieve tissue removal in a repeatable manner, it was essential to carbonize the tip of the fiber and to apply gentle pressure to ensure good contact between the fiber tip and NP tissue. Tissue removal using the laser was considered effective only when vapor and bubbles could be seen escaping through the needle. Furthermore, from preliminary post‐laser nuclectomy MRIs, fluoroscopy, and micro‐CT scans, it was not possible to identify and visualize the extent of laser damage and tissue carbonization within the disc. Thus, following the biomechanical tests, each specimen was transversely cut and measurements of the laser tract were made manually using digital calipers.

### 2.6 Quantifying the amount of nucleus material that was removed

The volume removed was determined through segmentation of the post‐nuclectomy MRIs (further details in the following section), while the mass removed was determined in both hydrated and dehydrated states. The hydrated mass was obtained by immediately weighing the material removed from the disc. The dry mass was obtained after dehydrating the material removed for 48 h in a fume cupboard. However, when using the laser, disc material was removed by ablation, carbonization, and evaporation, and thus, the mass removed was calculated from the change in mass of the specimen pre‐ and post‐surgery while considering the effect of evaporation on disc mass during the procedure.

Preliminary work showed that even for short periods of time, the specimen mass decreased linearly (*r* ^2^ = 0.9986) with time as the water evaporated from the tissue surface. To ensure that measured masses were due to laser ablation and not evaporation, the specimen was weighed (using scales with an accuracy of 0.001 g) every minute for a 10 min period both before and after laser treatment. Additionally, during the laser nuclectomy process, the mass was recorded at 400 J intervals. Two straight lines were then fitted (*r* ^2^ = 0.9986) to describe the loss of mass to the air in the 10 min periods before and after laser nuclectomy. The best‐fit lines were extrapolated to calculate the difference in masses obtained from the two lines at the beginning (t1) and end (t2) of the laser nuclectomy process. The average difference in mass between t1 and t2 was assumed to be the mass of tissue ablated. Preliminary work on a bovine specimen showed that this technique allowed masses to be measured within 98% when an item of a known mass was removed from the specimen.

### 2.7 Post‐nuclectomy imaging and image processing

Post‐nuclectomy scans enabled internal damage due to NP removal to be identified and the volume of material removed to be calculated. Any new dark areas or regions with hyperintense signals within the disc space were assumed to be locations of removed material, and as such was included when determining the volume of material removed. Regions of the hyperintense signal were considered to be fluid‐filled cavities that had been formed due to NP removal. The endplate was considered damaged if clear changes in the profile of the VB‐disc boundary were noted on the MRIs.

The volume removed was quantified by manually segmenting the region of interest within the disc space using Mimics. The software enabled a specimen specific 3D binary region‐of‐interest mask to be created. Since the volume measured was subject to intra‐observer error, this was investigated by the observer repeating the segmentation process on the same disc three times, on different days.

### 2.8 Location of volume removed

**FIGURE 3:**
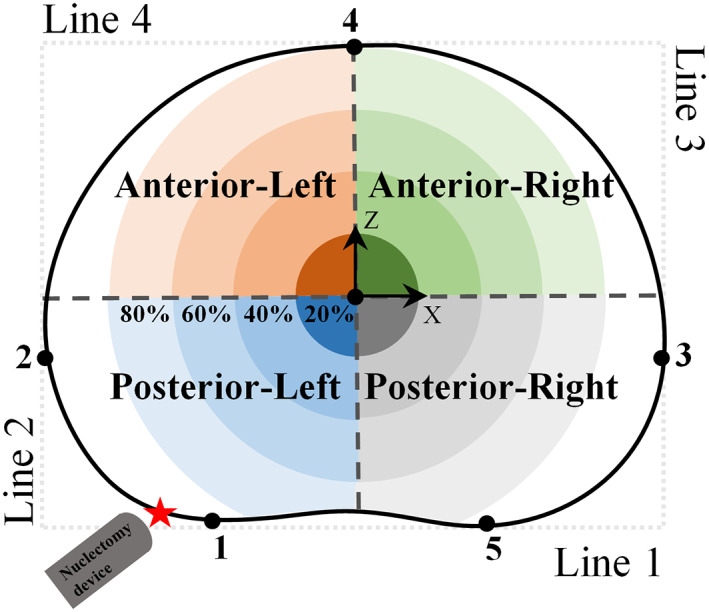
Disc centroids in the axial view were located using a method previously developed by Little et al.^65^ A rectangle was formed around the mid‐transverse plane of the IVD by drawing four lines. Line 1 passed through the two most posterior points on the AF boundary (Points 1 and 5). Line 2 and 3 were perpendicular to line 1 and passed through the lateral‐most points of the AF boundary (Points 2 and 3), and finally, line 4 was parallel to line 1 and passed through the most anterior point of the AF boundary (Point 4). The geometric center of the rectangle was used as the disc centroid from which concentric circular regions were drawn. The diameter of the concentric circular regions corresponds to percentage distances between Points 2 and 3. The red star represents the insertion point of the nuclectomy device

The locations from which the material was removed were determined using an adapted version of the method described by Little et al.^65^ to define the centroid of the disc in the transverse plane (Figure 3). Mimics was used to divide the disc into four quadrants with concentric circular regions about the disc centroid. The radii of the circular regions were determined by calculating 20%, 40%, 60%, and 80% of the largest lateral width of the disc which is between points 2 and 3 in Figure 3. The volume in each quadrant's circular sector was then measured. The volumes reported in the shaded regions in Figure 6 are calculated from the difference between two consecutive circular sectors in each quadrant.

### 2.9 Statistical analysis

All statistical analyses were performed using GraphPad Prism 9.3 (GraphPad Software Inc., San Diego, CA). Shapiro–Wilk's test was implemented to assess the normality of all statistical analyses, and whether the assumption of homogeneity of variance was legitimate was confirmed through Levene's test. Shapiro–Wilk's test confirmed the normality of the data (*p* > 0.05). Statistical significance of the reduction in stiffnesses between the intact state and post‐nuclectomy state were assessed using paired *t*‐test (95% confidence interval, significance level *p* = 0.05). Effects of nuclectomy techniques on volume and mass removed, size of AF incision, disc height reduction, and stiffness reduction between groups were assessed using one‐way ANOVA and post hoc Tukey tests at a 95% significance level. Pearson's *r* correlation was conducted to evaluate the association between the amount of material removed and the level of degeneration, toe and linear region stiffness change, disc height change, and volume of the cavity.

## 3 RESULTS

### 3.1 Volume and mass removed

**FIGURE 4:**
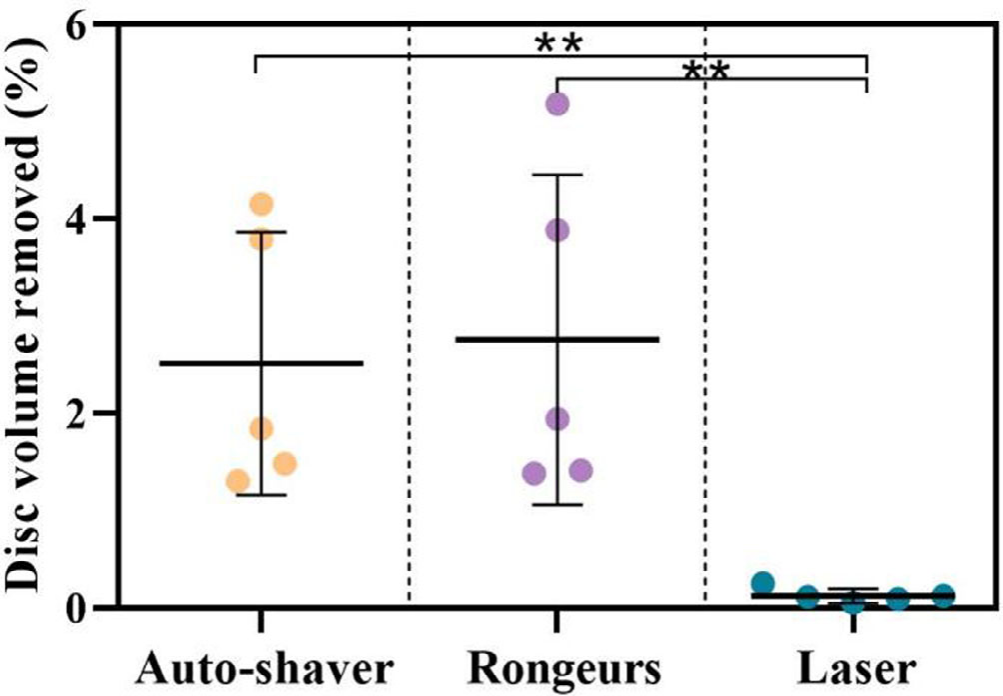
Average volume of disc material removed using each nuclectomy technique expressed as percentage of the whole intact disc volume. Double asterisks denote a significant inter‐technique difference

Nuclectomy using rongeurs removed more disc material (478.5 ± 129.4 mm^3^) than when either the automated‐shaver (353.3 ± 157.2 mm^3^, *p* = 0.75) or the laser (20.9 ± 12.4 mm^3^, *p* = 0.003) were used. The volume of material removed with respect to the intact disc volume are shown for each removal technique in Figure 4. Using rongeurs, a similar dry mass of disc material was removed as when using the automated shaver (0.254 ± 0.078 g and 0.229 ± 0.013 g, respectively; *p* = 0.54), however, the hydrated mass of disc material removed by the automated‐shaver was significantly more than that collected using the rongeurs (4.184 ± 1.310 g vs. 0.879 ± 0.207 g, *p* = 0.0011). Nuclectomy using laser resulted in the smallest change in hydrated mass (0.228 ± 0.038 g, *p* < 0.001). Post hoc analysis showed inter‐technique statistically significant differences among the normalized disc volumes removed, *p* < 0.027 (Figure 4). There were statistically significant correlations between the dry mass removed and volume of the cavity formed for the automated‐shaver and rongeurs groups (Pearson *r* = 0.933, *p* = 0.020 and *r* = 0.919, *p* = 0.027, respectively).

### 3.2 Comparison of pre‐ and post‐nuclectomy MRIs

**FIGURE 5:**
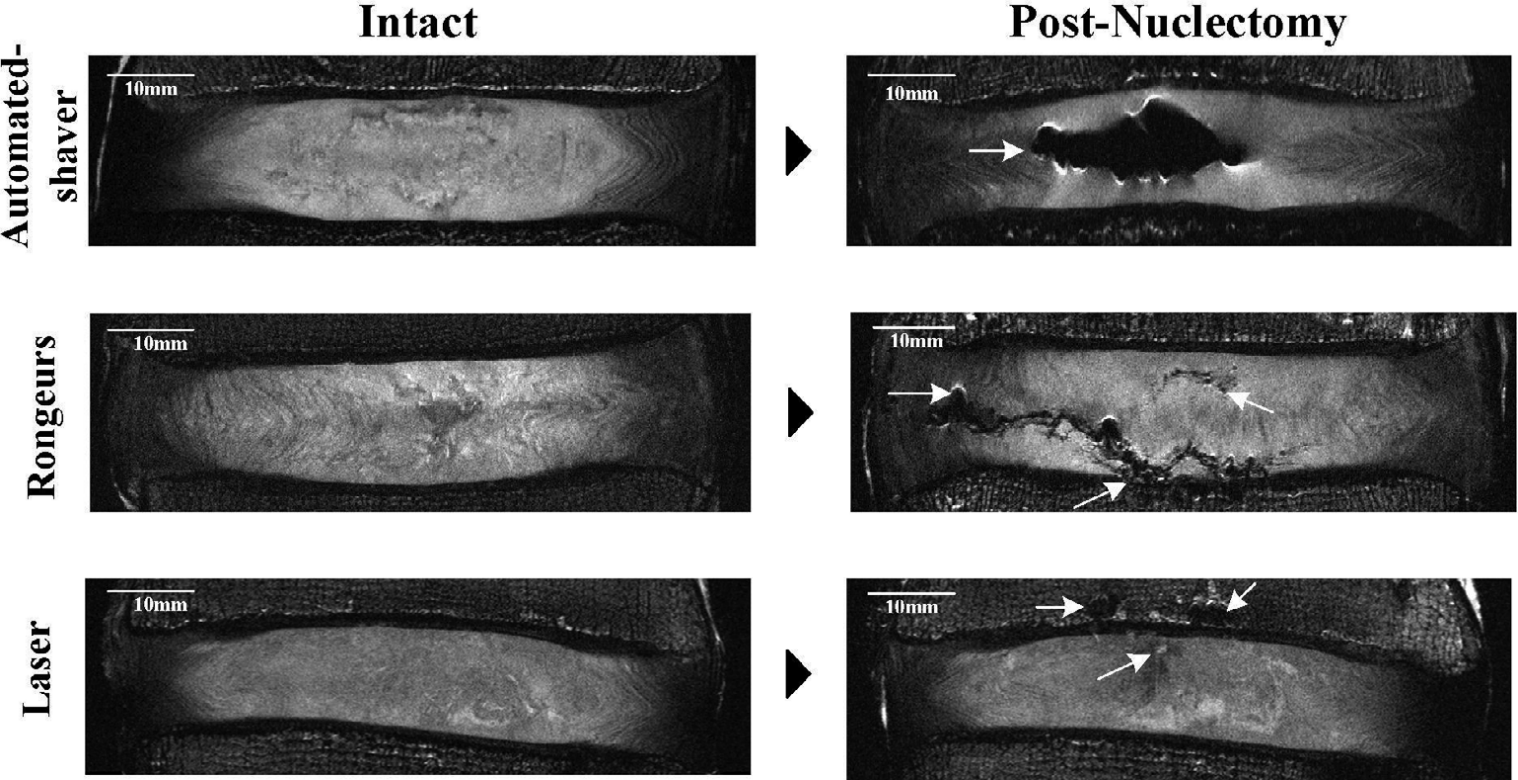
Typical coronal plane T2 weighted MRI of discs at both the intact and post‐nuclectomy stages. The white arrows indicate locations of damage. Using the automated‐shaver disc material was removed homogeneously approximately at the center of the disc (top row). Using rongeurs disc material was removed nonhomogeneously with some damage seen in the AF and at the endplates (middle row), and finally using laser, cavities were formed in the NP region that may have filled with condensed vapor, and cavitations were seen in the subchondral bone marrow (bottom row).

Pre‐ and post‐nuclectomy MRIs allowed visualization of typical cavities formed using each of the techniques (Figure 5). No changes were seen to the endplate profile in the MRIs from the automated‐shaver group, however, 60% of the specimens from the rongeurs group and 20% from the laser group had damage at the endplate in the form of small indentations, and 40% of the laser‐treated specimens showed changes of the subchondral marrow (Figure 5).

### 3.3 Location of volume removed

**FIGURE 6:**
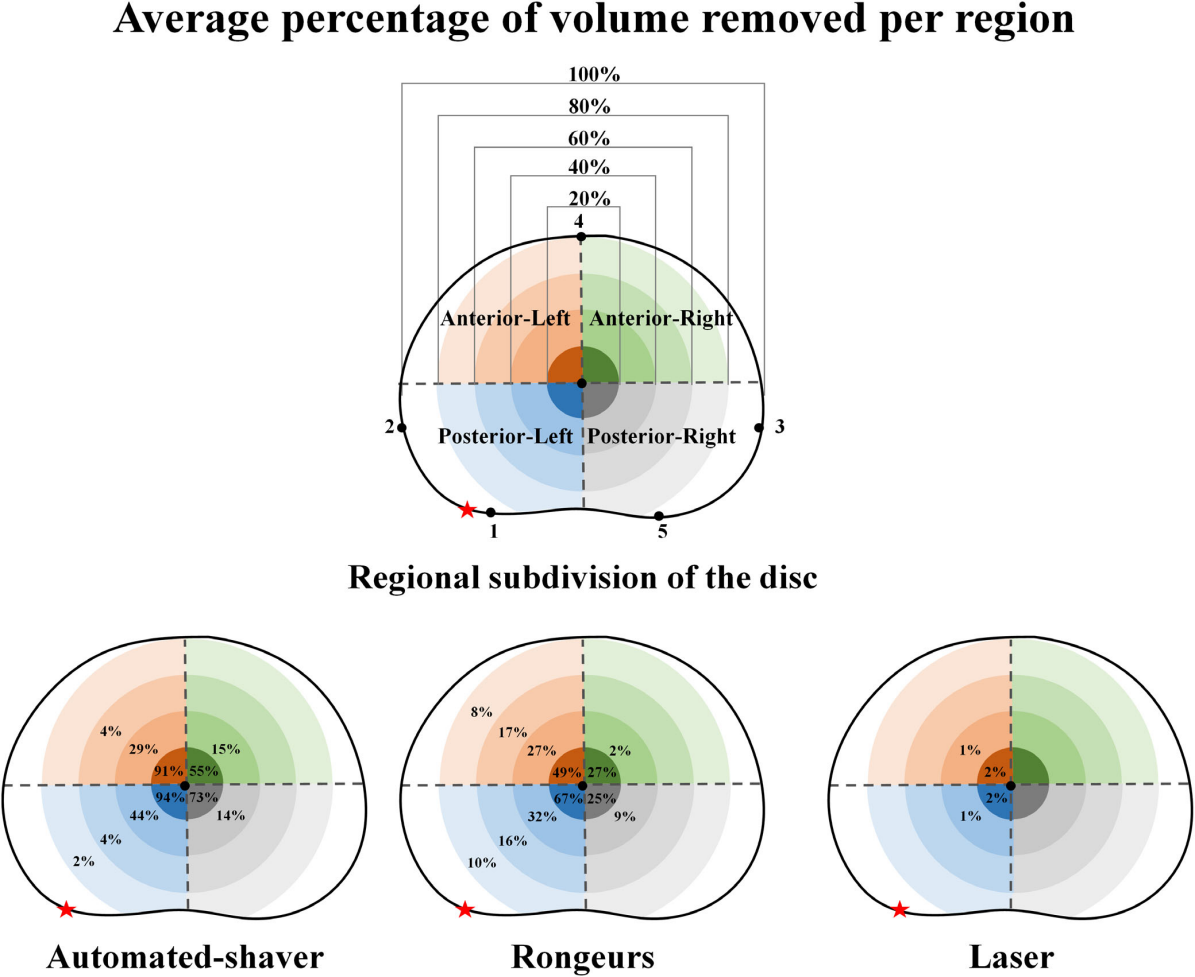
Transverse plane view of the disc showing the average percentage of volume removed with respect to each shaded region's intact volume using each of the three removal techniques. Regions with no numerical value indicate that no volume of material was removed. The diameter of the circular regions was determined by calculating 20%, 40%, 60%, and 80% of the largest lateral width of the disc calculated between Points 2 and 3. The red stars indicate the nuclectomy‐device entry location

When using the laser, the total volume removed was within the 40% boundary of the anterior–posterior (AP) region on the same side as the needle entry point. While when using the automated shaver and the rongeurs, between 70% and 80% of the total volume removed was in the AP region of the same side as the nuclectomy tool's entry point (Figure 6). When using the rongeurs, material was also removed from the outer regions of the disc (Figures 5 and 6). When using the automated shaver, disc material was removed homogeneously from the central region of the disc with less damage to the AF compared to when using rongeurs (Figures 5 and 6). In all techniques, over 75% of NP material was removed within the region that was less than 40% of the lateral width from the center of the disc, this was highest in the laser group where 100% of material removed was within this 40% boundary (Figure 6).

### 3.4 Size of annulotomy

Despite the initial incision through the AF being a single cut 3 mm in length, following nuclectomy the size of the entry point enlarged for both the rongeurs and automated‐shaver group's specimens. Both groups had similar final annular insult widths of 6.91 ± 1.50 mm for the rongeurs group and 6.84 ± 1.34 mm for the automated shaver group (*p* = 0.944). Meanwhile, the height of the final annular incision in rongeurs group was significantly larger than that of the automated‐shaver group(4.27 ± 0.70 and 2.34 ± 0.47 mm, respectively [*p* = 0.002]). When using the laser technique, the AF was only damaged via the single 18G needle puncture, hence caliper measurements were not possible.

### 3.5 Laser tract

**FIGURE 7:**
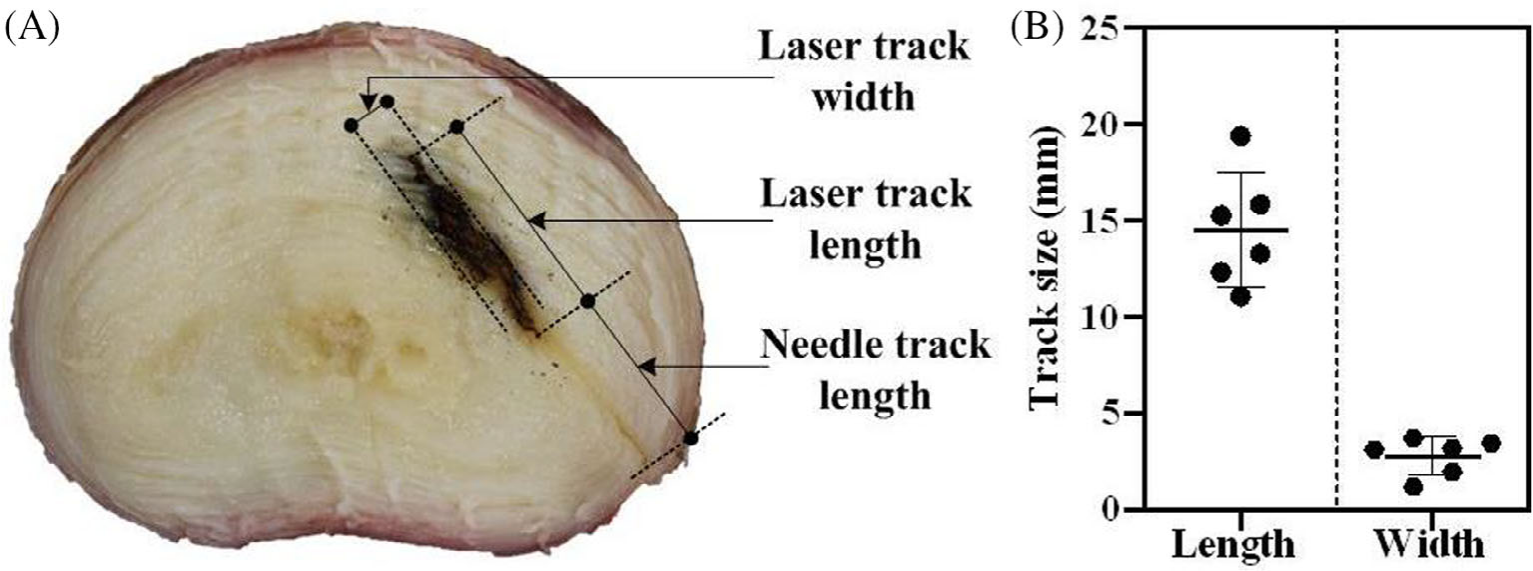
(A) Axial view of a transversely cut intervertebral disc following nuclectomy using laser. (B) Average laser track dimensions

The specimens from the laser group were cut transversely and carbonized tissue was visible in the area adjacent to the fiber insertion point (Figure 7). The average length and width of the laser track were 14.5 ± 0.39 and 2.75 ± 0.67 mm, respectively.

### 3.6 Stiffness

**FIGURE 8:**
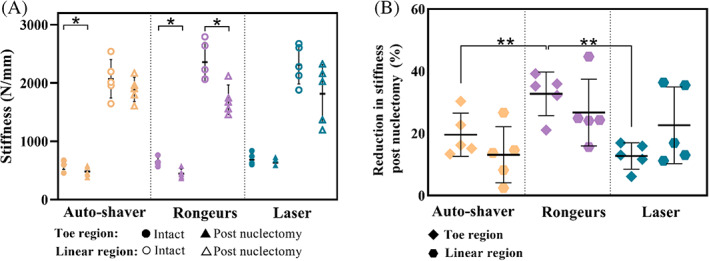
(A) Comparison of intact versus post‐nuclectomy stiffness values calculated from the force‐displacement graphs. Toe and linear region stiffnesses were calculated between 50–200 N and 500–900 N, respectively. (B) Normalized stiffness reduction in the toe and linear regions post‐nuclectomy. Single asterisks denote a significant reduction (*p* < 0.05) post‐nuclectomy compared to the intact state. Double asterisks denote a significant inter‐technique difference

For all specimens, post‐nuclectomy the stiffness reduced compared to intact state with a greater change in the toe region compared to the linear region for the automated‐shaver and rongeurs groups (Figure 8). A paired *t*‐test showed that the reduction in toe‐region stiffness between the post‐nuclectomy and intact state was statistically significant for both the automated‐shaver and rongeurs groups (*p =* 0.04 and *p =* 0.007, respectively), while the change in linear region stiffness between the post‐nuclectomy and intact states was significant only for the rongeurs group (*p =* 0.011). The reduction in toe‐region stiffness between the intact and post‐nuclectomy tests in the specimens subjected to NP removal using the rongeurs was significantly different than those from the automated shaver and laser groups (*p* < 0.0054). Only in the automated‐shaver and rongeurs groups were statistically significant relationships between dry mass removed and toe region stiffness found (Pearson *r* = 0.948, *p* = 0.014 and *r* = 0.915, *p* = 0.029, respectively).

### 3.7 Disc height

**FIGURE 9:**
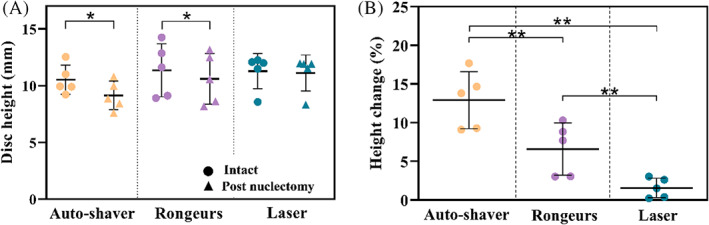
(A) Central coronal disc height measurements in the intact and post‐nuclectomy states. (B) Percentage reduction in central disc height compared to the intact discs. Single asterisk denotes significant disc height reduction. The double asterisks denote a significant inter‐technique difference (*p* < 0.05)

A greater reduction (*p* < 0.02) in average disc height was seen following nuclectomy with the automated shaver compared to the rongeurs, 1.4 ± 0.3 mm versus 0.74 ± 0.3 mm, respectively (Figure 9A). Laser nuclectomy resulted in the smallest reduction in disc height (0.2 ± 0.1 mm, *p* = 0.46). Statistical significances in disc height changes were found between all three the nuclectomy techniques (*p* < 0.038, Figure 9B). A significant relationship between the dry mass removed and the change in disc height was only seen in the automated shaver group (Pearson *r* = 0.82; *p* = 0.09).

## 4 DISCUSSION

This study compared three techniques that have previously been found to have various degrees of clinical utility to remove tissue from the central region of an intervertebral disc for decompression, and more recently prior to nucleus replacement.

Although discectomy of herniated lumbar discs has been successful in relieving pain in the short term, studies have shown that limited nucleus removal is associated with higher risk of reherniation, while more aggressive NP removal is associated with accelerated disc degeneration and increased LBP due to spondylotic changes of the spinal segment.^20^, ^21^, ^22^ Therefore, the quantity of disc material to be removed is a matter of debate, as clinical studies have not found a correlation between the amount of material removed and long‐term outcome.^14^, ^66^, ^67^, ^68^

### 4.1 Intradiscal void post‐nuclectomy

Although nuclectomy using the automated shaver and rongeurs removed similar volumes of material (*p* = 0.75), the way in which tissue removal was achieved differed. The automated shaver removed disc material via a vacuum tissue removal system that aspirated the disc material cut by the small side‐port close to the tip of probe. Both the firm structure of the AF and the blunt tip of the probe ensures that predominantly NP tissue was removed.^69^ This resulted in homogeneous cavities in the central region of the disc, as shown in Figure 5, which might be suitable for both pre‐formed and in situ forming nucleus replacement devices. The small indentation at the tip of the rongeurs allowed tissue to be grasped and torn away from its surroundings. This resulted in a relatively invasive approach where it is possible for AF material to be removed inadvertently, creating the inhomogeneous cavities seen in Figure 5 which were also observed in another *in vitro* trial by Brinckman and Grootenboear.^70^

Laser nuclectomy resulted in the least amount of material removal (20.9 ± 12.4 mm^3^). Lasers have been used for percutaneous lumbar disc decompression (PLDD) to treat herniations.^37^ Tissue was charred and vaporized by the optical fiber as it was advanced into the disc (Figure 7), and only disc material that was adjacent or local to the fiber tip was ablated. This resulted in minimal tissue removal suggesting that the laser parameters reported in previous studies are not suitable for the ablation of large volumes of NP material. In the MRIs of the post‐laser nuclectomy discs, regions of signal hyperintensities within the NP were observed (Figure 5). These suggest that the voids formed due to ablation might have been filled with liquid. Gas‐filled cavities were also seen in one specimen recognized by the sharply defined edges which were also reported by Gangi et al.^71^ and Schenk et al.^72^ Choy et al.^37^ reported removing between 31.46 and 96.21 mm^3^ of disc material through laser ablation. Their volumetric calculations were made by assuming that the laser track is cylindrical (Figure 7). Based on this assumption the theoretical volume of material removed in this study would be within the range reported, 88.20 ± 52.82 mm^3^. The discrepancy between this theoretical volume and the one reported following the segmentation of cavities seen on the MRIs (20.9 ± 12.4 mm^3^) is likely due to internal pressure within the IVDs causing the tissue to redistribute and reduce the size of any newly formed cavities.

Complete nuclectomy using lasers may be possible if the amount of energy delivered was increased however caution must be applied since this may result in high temperatures within the IVD which would damage the surrounding tissues.^73^, ^74^ Alternatively, shorter but higher power pulses followed by 5–10 s pauses have the potential to remove larger volumes of tissue without collateral damage.^72^, ^75^, ^76^, ^77^ Further research is required to determine the optimal laser parameters for this application.

### 4.2 Annular incision

The initial AF incision was made such that it was just large enough for the 3 mm automated‐shaver probe or rongeurs to enter into the disc space. Following nuclectomy using the automated shaver and the rongeurs, the width of the insult had doubled (6.91 ± 1.50 mm vs. 6.84 ± 1.34 mm, respectively) due to maneuvering the tool to maximize disc tissue removal. However, the height of the AF insult was significantly higher for the rongeurs group compared to the automated‐shaver group (4.27 ± 0.70 mm vs. 2.34 ± 0.47 mm, *p* = 0.002). When using the automated shaver, to maximize tissue removal, the probe was moved in a fan‐shaped pattern while also being rotated around its own axis as the tissue was aspirated away from the disc space. Meanwhile, when using the rongeurs, the tools had to repeatedly pass through the annulotomy resulting in an enlargement of the annular incision. Out of the three techniques, laser nuclectomy was the least disruptive to the annulus as it required a single 18G needle puncture to insert the fiber into the disc space. The advantage of a smaller annulotomy is that it might minimize implant expulsion, which has been a challenge from the onset of nucleus replacement surgeries.^11^, ^78^ However, it should be noted that the risk of expulsion may also be dependent on other factors such as the ability of the nucleus replacement device to adhere to surrounding tissues, and gelation times once inserted into the IVD.^79^

### 4.3 Endplate damage and morphological changes

Internal morphological differences could be observed from the post‐nuclectomy MRIs in the form of damage to the endplate, subchondral marrow changes, and altered signal intensity from the NP. Endplate damage was seen in 60% and 10% of discs that were subjected to nucleus removal using rongeurs and laser‐nuclectomy, respectively (Figure 5). This is particularly important as it can affect disc biomechanics, such as changing stiffnesses (Figure 8) and load distributions.^26^, ^80^, ^81^, ^82^ However, the clinical significance of endplate damage and its association with LBP and accelerated degeneration is still limited by the current diagnostic tools.^83^, ^84^ Endplate damage may affect the supply of nutrients to the disc, which could not be assessed through this *in vitro* study but warrants further investigation, particularly since the long‐term effects of endplate damage on the health of the AF in the absence of a native NP is not well understood.

Subchondral bone marrow changes were observed in two of the five discs in the laser nuclectomy group. It is speculated that subchondral abnormalities are due to thermal damage as a result of energy penetration into the bone when the laser fiber is positioned close to the endplate.^85^, ^86^, ^87^ Cvitanic et al.^87^ and Kosaka^85^ report that clinically the subchondral bone marrow changes resolve within 5–7 years.

Tissue carbonization was not seen on the MRIs but was observed in all discs once transversely cut (Figure 7A). From Figure 7A, it can be noted that when using the laser, AF material can inadvertently be removed, depending on the positioning and movement of the needle within the disc space. Similar results have been reported by Choy et al.,^37^ Schenk et al.,^72^ and Ferreira et al.^42^ suggesting that specifically targeting the NP using this method may be challenging. The laser track lengths and widths reported in this study (14.50 ± 0.39 and 2.74 ± 0.67 mm, respectively) were similar to those reported by Choy et al.^37^ (10 and 2–4 mm, respectively) but considerably larger than the 0.94–2.23 mm lesions reported by Plapler et al.^77^ who instead kept the fiber tip at a single location. It was noted that laser nuclectomy did not provide good tactile feedback during the procedure compared to using the automated shaver or rongeurs. Therefore, care to measure the size of the target disc prior to surgery to ensure that the fiber tip is not inadvertently advanced through the AF is advised.

### 4.4 Stiffness

The NP is thought to withstand much of the stresses at lower loads with a shift to AF‐dominated support at higher loads.^88^, ^89^, ^90^, ^91^ The larger reduction in toe‐region stiffness compared to linear‐region stiffness when using the automated shaver and rongeurs in this study (Figure 8C) reinforces this theory.

The observed decrease in stiffnesses (overall average of 34.9% and 17.4% in the toe and linear regions, respectively) is within 3%–50% of the range reported by the previous studies.^90^, ^92^ The significant difference between the average toe‐region stiffness of the rongeurs group compared to the automated‐shaver and laser groups may be explained by the substantial collateral damage caused by the rongeurs to the surrounding tissues (Figure 6). This relatively crude technique also resulted in damage to AF regions that may have resulted in the significant decrease in linear region stiffness that was also reported by Showalter et al.^92^

Laser nuclectomy did not significantly affect the stiffness of the disc; however, the small reduction in stiffness may be attributed to either depressurization of the disc,^93^ internal chemical changes caused by tissue carbonization, or to disruption caused to the AF when inserting the needle.^82^ A comparative study between partial and more aggressive nuclectomy conducted by Cannella et al.^90^ has shown that removing as little as 0.05 g of dry NP material significantly affects disc height, stiffness, compressive and tensile range of motion, and intradiscal pressure. Removing more than 0.2 g of dry NP material significantly increases the neutral zone response of the disc.^90^ The effect of nucleus removal on increased disc instability has also been confirmed by multiple computational models.^94^, ^95^, ^96^

### 4.5 Disc height

Brinckman and Grootenboear^70^ reported a 0.8‐mm decrease in unloaded disc height for every gram of hydrated disc material removed. This correlation gave estimate values within 5% of the actual disc heights measured from the post‐nuclectomy MRIs for the rongeurs (0.74 ± 0.3 mm) and laser (0.2 ± 0.1 mm) groups in this study. The automated‐shaver group reported the largest reduction in disc height (13.2 ± 1.6%, *p =* 0.001) compared to rongeurs (6.6 ± 1.2%, *p =* 0.016) and laser (0.7 ± 0.1%, *p =* 0.12) groups (Figure 9). According to the Brinckman and Grootenboear^70^ disc height versus material removed relationship, the average expected unloaded disc height reduction for the automated‐shaver group would have been approximately 3.2 mm which is considerably greater than the 1.4 ± 0.3 mm change measured from the MRIs. This difference is likely due to the NP tissue removed with the automated shaver being more hydrated than in its physiological state and the disc heights in this study being measured under 50 N axial load (rather than unloaded). Additionally, although a linear relationship between disc height and the amount of material removed was reported by Brinckman and Grootenboear,^70^ a single needle puncture alone (i.e., no material removed) has been shown to be sufficient to initiate disc height loss demonstrating a possible limitation of this relationship.^82^, ^97^

The volume removed (478.5 ± 129.4 mm^3^) and disc height loss (0.74 ± 0.3 mm) seen in the rongeurs group in this study were toward the higher end of the values reported by Zengerle et al.^32^ (150–300 mm^3^ and 0.3–0.7 mm) although their measurements were made with 100 N of compression on the specimen, rather than the 50 N used here. The range of the disc height reduction seen in the post‐laser group in this study, 0.2 ± 0.1 mm was similar to that reported by Kutschera et al.^98^ in the unloaded state (0.1–0.32 mm). The fact that disc height loss values were comparable to those reported by others, gives confidence that the techniques have been accurately replicated for the purposes of this study.

### 4.6 Mass removed

Analyzing the hydrated masses, the automated shaver evacuated more disc material than the rongeurs (4.184 ± 1.310 g and 0.879 ± 0.207 g, respectively, *p* = 0.001). This is likely due to the automated shaver using an irrigation system to remove the cut material from the disc, causing NP tissue to be more hydrated than in its physiological state. The hydrated mass removed using the automated shaver was within the 1–7 g range reported in other studies^99^, ^100^ while the dry mass removed (0.229 ± 0.013 g) was similar to the 0.24 g reported by Cannella et al.^11^ The hydrated mass of material removed when using the rongeurs in this study (0.879 ± 0.207 g), is less than the average values reported by Showalter et al.^92^ and Smith et al.,^101^ 1.71 and 1.64 g, respectively. This is possibly due to the larger rongeurs used by Showalter et al.^92^ and Smith et al.^101^ (4 mm vs. 3 mm in this study).

Although long‐term clinical outcomes following disc decompression treatment using lasers have been widely studied,^37^, ^38^, ^72^, ^102^, ^103^, ^104^, ^105^, ^106^, ^107^, ^108^, ^109^ the volume or mass of NP material removed is seldom reported, likely due to difficulties in measuring these parameters *in vivo*. In studies that have attempted to quantify mass and volume of the removed NP material, methods differ making like‐for‐like comparisons challenging. In cadaveric specimens, a common method is to weigh the specimen before and after laser ablation of disc material. Using this method, a range of 0.2–0.25 g of wet NP material has been reported to be removed,^63^, ^110^, ^111^ which is similar to the mass removed in our study (0.228 ± 0.037 g). For the laser nuclectomy specimens, it was not possible to measure dry mass due to the nature of the procedure, however considering the ages of the specimens in the laser group it can be assumed that the water content of the NP is approximately 70%,^112^ and therefore the dry mass removed would have been approximately 0.069 ± 0.011 g, which is significantly lower than the dry mass removed using the rongeurs and automated shaver (*p* < 0.01).

Results of this and other studies have shown that changes in disc biomechanics are affected by the amount of material removed.^70^, ^90^, ^113^, ^114^ Thus, to allow comparison of the masses removed using the different nuclectomy techniques, the excised wet mass needs to be converted to a dry mass, to remove the confounding variability due to disc hydration. Furthermore, regardless of the technique used to achieve NP removal, clinical studies have not found a correlation between the amount of material removed and the long‐term outcomes of nuclectomy, thus the appropriate quantity of disc material to be removed is still unclear.^14^, ^66^, ^67^, ^68^

### 4.7 Limitations

For consistency, a single surgeon carried out the nuclectomies, however, like any other surgeries, there is a learning curve to nuclectomies. Thus, further investigation into the correlation between the surgical skill level and outcomes in terms of collateral damage, material removed, and location of tissue removal is warranted. Despite the small number of specimens per group (*n* = 5), the findings from this study may be cautiously extrapolated to clinical applications. However, in clinical settings, the extent of disc material removal might be less pronounced if straight shaver probes and rongeurs are used as the spinal posterior elements would likely restrict the maneuverability. This could possibly be overcome by using a curved shaver probe or angled rongeurs. Additionally, 10 W pulses were delivered to ablate disc material using the laser, this parameter was at the lower end of the range of powers that have been used in other studies (10–20 W),^37^, ^38^, ^41^, ^62^, ^63^, ^98^ which likely means that the volume and mass of tissue removed are underestimations of what could potentially be removed.^63^

Although the cut face of the vertebrae was pushed up against the rigid pot surface before being fixed in PMMA to ensure the load paths through the vertebrae were as realistic as possible, the lack of a full vertebrae may mean that the stiffness values obtained here were not a true representation of the *in vivo* response. However, since all samples, in all states (intact and post‐nuclectomy) were potted in this way, the peak loads were relatively low (1 kN) and not close to failure, and the calculated stiffnesses were within the range reported in literature (2226 ± 304 kN/mm in this study vs. a range of 1734–3862 kN/mm reported by Newell et al.^115^ and Beckstein et al.^116^) comparisons between groups are still valid. Additionally, despite ensuring that disc heights were >5 mm, some of the specimens were potentially towards the later stage of degeneration (Pfirrmann's grade 4)which could have affected the amount of material removed due to tissue becoming more fibrous.^117^

Limitations such as donor age, degeneration grade, and overall disc health may have affected the location and amount of material removed, however investigating their effect on the outcomes of the nuclectomies was out of the scope of this study. Disc degeneration is known to affect disc biomechanics,^80^, ^118^ but no correlation was found between Pfirrmann grade and the change in stiffness post nuclectomy here. A future study with a larger sample size per group may allow these correlations to be investigated further.

## 5 CONCLUSION

This study investigated three different surgical techniques that have been used clinically to remove nucleus material. The three nuclectomy techniques were compared by acquiring ultra‐high resolution MRIs to noninvasively visualize the extent of damage and location of tissue removal, and by investigating changes in disc height and response to load. Currently, nucleus replacement surgeries are not routinely used due to the lack of clinically viable technology. Determining an effective way of removing NP material that is least damaging to the surrounding structures, is an important step to develop the next generation of nucleus replacement surgeries.

The results have shown that out of the three techniques used in this study, the automated shaver is better suited for nuclectomy as damage to surrounding structures is limited to the central region of the disc and changes to the disc's linear region response are smaller, suggesting that the AF was less damaged. Further evidence to support the results of this *in vitro* study would be beneficial to select the most appropriate technique for nuclectomy.

## AUTHOR CONTRIBUTIONS

Tamanna Rahman and Nicolas Newell designed the study. Nicoleta Baxan, Tamanna Rahman, and Nicolas Newell designed sequences and obtained the MRI images. Jonathan Bull carried out nuclectomy using rongeurs and the automated shaver. Robert T. Murray carried out laser nuclectomy. Thomas P. Schaer lent the automated shaver. Tamanna Rahman and Saman Tavana analyzed the data and drafted the manuscript which was edited by Nicolas Newell, Nigel Smith, Robert T. Murray, Nicoleta Baxan, Thomas P. Schaer, and Saman Tavana. The manuscript has been approved by all authors before submission.

## FUNDING INFORMATION

Part of this work was funded by an Imperial College Research Fellowship for Nicolas Newell and an EPSRC DTP CASE Conversion Studentship for TR (EP/R513052/1).

## CONFLICT OF INTEREST

The authors declare that the research was conducted in the absence of any commercial or financial relationships that could be construed as a potential conflict of interest.
